# Chasing the dragon - characterizing cases of leukoencephalopathy associated with heroin inhalation in British Columbia

**DOI:** 10.1186/1477-7517-8-3

**Published:** 2011-01-21

**Authors:** Jane A Buxton, Renee Sebastian, Lorne Clearsky, Natalie Angus, Lena Shah, Marcus Lem, Sian D Spacey

**Affiliations:** 1 Epidemiology Services, British Columbia Centre for Disease Control, Vancouver, BC, Canada; 2 School of Population and Public Health, University of British Columbia, Vancouver, BC, Canada; 3 Canadian Field Epidemiology Program, Public Health Agency of Canada, Ottawa, ON; 4 School of Public Health University of Saskatchewan, SK, Canada; 5 Division of Neurology, Dept. of Medicine, University of British Columbia, Vancouver, BC, Canada

## Abstract

An association between leukoencephalopathy, a disease of the white matter of the brain, and smoking heroin is well recognized. This paper describes 27 cases of leukoencephalopathy identified in two cities in British Columbia, Canada 2001-2006; the largest number of geographically and temporally defined reported cases in North America.

Twenty cases of leukoencephalopathy were identified in and around Vancouver with onset dates December 2001 to July 2003; seven further cases were identified in Victoria September 2005-August 2006. Twenty (74%) of all cases were male, two couples were reported and eleven cases (55%) had Asian ethnicity. One case reported smoking heroin on a single occasion and developed mild symptoms; all other cases were hospitalized. Thirteen (48%) cases died; all had smoked heroin for a minimum of 3 years. Testing of one available heroin sample identified no substance other than common cutting agents.

Although a specific etiology was not identified our study supports the theory of an intermittent exposure to a toxic agent added to the heroin or a combustion by-product. It also suggests a dose response effect rather than genetic predisposition. Collaboration with public health, health professionals, law enforcement and persons who use illegal drugs, will facilitate the early identification of cases to enable timely and complete follow-up including obtaining samples. Testing of implicated heroin samples may allow identification of the contaminant and therefore prevent further cases. It is therefore important to ensure key stakeholders are aware of our findings.

## Introduction

Leukoencephalopathy refers to disease of the white matter of the brain and therefore can involve motor, sensory, and visual systems. Leukoencephalopathy can also disrupt cognitive and emotional function. There are many etiologies of leukoencephalopathy, including genetic disorders, cerebrovascular disease, eclampsia and toxic exposures. The clinical manifestation of the disease is a reflection of the areas of the brain involved in the disease process. Clinical features range from inattention, forgetfulness and personality changes, to dysarthria, ataxia, dementia, coma and death [1]. Toluene, ethanol, cocaine, methylenedioxymethamphetamine (MDMA or "ecstasy") and heroin have all been associated with toxic leukoencephalopathies [1]. Toxic exposure from heroin induced leukoencephalopthy typically involves the occipital lobes and cerebellum bilaterally, characteristic symmetric patterns can be seen on neuroimaging [2-5].

An association between leukoencephalopathy and smoking heroin has been recognized for over 25 years, although the exact pathogenesis is still not well understood. In 'chasing the dragon', heroin is placed on a piece of aluminium foil, heated with a flame from below, and the resulting vapour (pyrolysate) is inhaled with a straw or other tube-like structure. The practice was first recognized in Hong Kong in the 1950's but has now spread to users worldwide [6].

Although the practice of 'chasing the dragon' is not uncommon, the associated leukoencephalopathy has been rarely reported. The first and largest outbreak of leukoencephalopathy linked to chasing the dragon was reported 1982 in the Netherlands, and included 47 cases, 11 (23%) of whom died [6]. Since 1982, sporadic cases and small case clusters (ranging from 1-4 cases) have been report ed in Taiwan [7,8], Hong Kong[9], other European countries [4,5,10-13], the United States [3,14,15] and Canada [16]. Three larger clusters have been identified in China [17-20]. The primary hypothesis is that leukoencephalopathy is caused by a contaminant in the heroin or a combustion by-product[6,21,22]. Despite multiple attempts to identify a contaminant in heroin samples; no causative agent has yet been identified.

Between December 2001 and July 2003, 20 cases of leukoencephalopathy linked to heroin inhalation were identified in and around Vancouver, British Columbia (BC) Canada. An investigation was initiated at the time; but no causative agent was identified. Case reports were created to describe the clinical, pathologic, and imaging findings and awareness campaigns were initiated.

In the fall of 2005, further cases of leukoencephalopathy were reported in Victoria, BC on Vancouver Island. An investigation was initiated in order to describe the epidemiology of the new cases. The purpose of this paper is to characterize all cases of heroin associated leukoencephalopathy identified since 2001 to date in BC in order to guide future research and public health actions.

## Methods

A case of heroin associated leukoencephalopathy was defined as:

A person with clinical features of toxic leukoencephalopathy +/- neuroimaging with white matter changes typical of heroin-associated leukoencephalopathy

AND a history of chasing the dragon

AND a resident of BC or reported obtaining heroin in BC

A case report form (which collected demographic, drug use, and clinical information) and a fact sheet for physicians and the public were developed. The Provincial Health Officer notified BC neurologists about the cases of leukoencephalopathy associated with 'chasing the dragon' through the provincial specialty society list serve. Medical Health Officers throughout the province were also informed; they in turn notified the hospital Emergency Department heads and family physicians in their health regions. All physicians were requested to notify cases to local public health, which ensured the completed case report form was sent to the BC Centre for Disease Control.

Where possible, charts of cases were reviewed and interviews were conducted with the case or next of kin. Abstracted information included: sex, date of birth, residence and ethnicity, medical, social and drug histories, date of symptom onset, date of hospital admission, clinical course and outcome. Cases were asked if a heroin sample was available for testing. A process was arranged for samples to be transported by the police to the Health Canada Drug Analysis Laboratory for content analysis using liquid chromatography-mass spectrometry.

## Results

**Figure 1:**
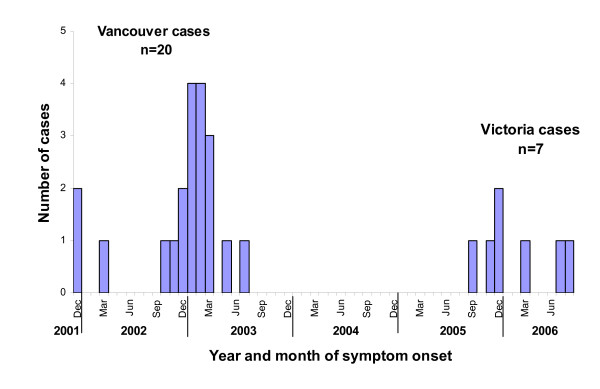
**Leukoencephalopathy cases associated with chasing the dragon in British Columbia, 2001-2006 by symptom onset date (n = 27)**.

In addition to 20 cases identified in and around Vancouver with onset dates between December 2001 - July 2003, a further 7 cases were identified in Victoria with onset between September 2005- August 2006 (Figure 1). All cases, except one, were hospitalized. Thirteen (48%) died and 20 (74%) were male (Table 1). Two heterosexual case-couples were reported; one pair in Vancouver and one in Victoria. Eleven Vancouver cases (55%) reported Asian ethnicity; while all Victoria cases were Caucasian.

**Table 1:** Demographic and outcome variables among leukoencephalopathy cases in BC (n = 27)

Variable	Vancouver (n = 20)		Victoria (n = 7)		All cases (n = 27)		Vancouver vs. Victoria
	**Mean**	**Range**	**Mean**	**Range**	**Mean**	**Range**	**P-value**^†^

**Age (years)**	36	32 - 42	32	21 - 51	33	21 - 51	0.220

	**N**	**%**	**N**	**%**	**N**	**%**	**P-value**^‡^

**Sex: Male**	15	75	5	71	20	74	1.000
**Ethnicity: Asian**	11	55	0	0	11	41	0.022*
**Hospitalized**	19	95	7	100	26	96	1.000
**Deceased**	10	50	3	43	13	48	1.000

^† ^P-value determined using t-test.

^‡^P-value determined using Fisher's exact test.

*Statistically significant difference (p < 0.05)

Patients typically presented with symptoms of cerebellar dysfunction such as ataxia, and all cases reported difficulty with speech. The clinical and imaging findings of three of the Vancouver cases are presented elsewhere [2]. The date of death was available for seven of the deceased, for these the median time between symptom onset and death was 54 days (range: 16-408 days).

Drug histories were obtained for 18 (67%) cases. One case that reported smoking on a single occasion developed mild symptoms and was not hospitalized. Excluding this case the mean duration of chasing the dragon was 9.5 years (range 0.5 - 30 years). Of the cases that died, the minimum time of smoking heroin was three years. Use of other illicit drugs was reported in both Vancouver and Victoria cases (cocaine, marijuana, ecstasy, and crystal methamphetamine). Three (43%) of the Victoria cases reported that smoking heroin was their only type of illicit drug use. At least six (86%) of the Victoria cases were taking methadone at the time of symptom onset; and five (25%) Vancouver cases reported using methadone, although these data are incomplete.

Drug supply information was available for four (57%) of the Victoria cases; all reported obtaining heroin through telephone order and home delivery (dial-a-dope) from a male Asian supplier. The first three Vancouver cases also reported being supplied by an Asian supplier through dial-a-dope but data are incomplete for the rest of Vancouver cases.

Three (47%) of the Victoria cases reported commercial painting as an occupation. No associations were found with type of aluminium foil (commonly purchased at the local supermarket); or with other underlying conditions or medication other than methadone. No difference in colour, texture or smell of the heroin was reported by the cases. Apart from the two couples no cases reported knowing anyone else with similar symptoms. One heroin sample from a Vancouver case was tested with only common cutting agents identified.

## Discussion

We have characterized 27 cases of leukoencephalopathy associated with 'chasing the dragon'. This is the largest number of cases reported in North America which are temporally and geographically defined. Although an etiologic agent has not been identified, we have a better understanding of the population at risk. The age, sex and ethnicity of our cases are consistent with the demographic profiles in other published reports. Forty-one percent of BC cases were Asian; this preponderance, also found in other studies, is likely representative of persons who 'chase the dragon' [6].

Identification of two heterosexual case-couples suggests that the risk factors for leukoencephalopathy are more likely to be substance related rather than due to genetic predisposition. The distribution of the cases in place and time suggests a common intermittent exposure. Substances added to the heroin may be an inert 'cutting agent' such as caffeine, lactose or mannitol to increase the volume and hence profit, or an 'adulterant', which is added for its pharmacological effect [23]. The dial-a-dope delivery system identified by some cases may involve additional persons in the supply and delivery chain (i.e., from the dealer to the deliverer) and increase the risk of contaminants being added, either for profit or to retain some drug for personal use. It is unlikely that the contaminant is added to cause intentional harm as it is in the best interest of the dealer to maintain his/her client base[24].

According to a recent report from the UN Office of the Drug Commission, 96% of heroin seizures (2002-2007) in the US originated from Mexico and Columbia; whereas 98% of heroin seized in Canada originated from Southwest Asia [25]. Although the source of heroin differs between Canada and US, and the epidemiology and prevalence of 'chasing the dragon' in BC is poorly understood, the incidence and risk of the resultant leukoencephalopathy is clearly low. We believe this indicates the contaminant is likely added close to the final delivery stage, rather than at the original source. However there is likely under-reporting as physicians are required to actively report the condition to public health, some cases may have been mild and spontaneously recovered and others attributed to other etiologies.

The purity of street heroin in BC, determined by Health Canada, Drug Analysis Service, has increased from 5-10% in the 1970's, to greater than 60% [26]. However, heroin used for smoking is usually 30% to 40% pure as higher grade cuts char too quickly for effective smoking. Heroin is also reported to be increasingly available in the base form which is not amenable for injection [27]. Smoking heroin in North America was becoming established prior to the knowledge of the risks of HIV associated with injecting [28]. Gossop *et al *found that chasing the dragon was a well-established method of using heroin in certain populations, not merely a pre-injection phase of heroin addiction [29]. Therefore, drug availability, attitudes to using needles, stigma and the potential of disease transmission related to injection drug use may have led to increased smoking rather than injection of heroin. This illustrates the potential for further cases to occur.

One case with a single brief exposure to inhaling heroin pyrolysate required outpatient support only. This finding was similar to a case report in the literature of a patient with an isolated exposure who had a complete recovery, and is consistent with a dose-response relationship [3,12]. Clarity of quantity and purity of heroin used by cases would allow a better understanding of a dose-response relationship.

Reported clusters of leukoencephalopathy have been associated with smoking heroin. However isolated cases of leukoencephalopathy associated with using heroin and heroin and cocaine intravenously [30-32], and one case of leukoencephalopathy associated with heroin ingestion occurring in a 2 year old child have been reported [33]. The development of disease associated with other routes of administration highlights the lack of knowledge about the etiological agent and the importance of determining its identity.

The etiology of heroin-related toxic leukoencephalopathy requires further research and public health involvement. The severity of the outcome and lack of curative treatment highlights the importance of future investigations. Current therapy with coenzyme Q and vitamin supplements is anecdotal only [34]. Previous literature has been mostly published in neurology and radiology journals as clinical case reports; isolated cases make it difficult to determine risk factors for this condition. Research into the prevalence of 'chasing the dragon' will help determine the potential risk for further outbreaks and may indicate a need to modify both educational, treatment and support services for this group of heroin users. Although a specific etiology has not been identified, a toxic agent added to the heroin, or a combustion by-product, remain the leading theories [6,21,22].

Limitations of our study include incomplete case and drug information. This may be related to the inability of cases to communicate at presentation and the rapid decline in mental state of some. Also, the illicit nature of drug use may cause concerns about sharing information regarding other drug users or the source of heroin. Family members may have little prior knowledge of the case's drug use, limiting the information provided through collateral history. The delay between heroin use and symptom onset also reduces the likelihood that implicated heroin is available for testing.

Recognizing the difficulties inherent in studying such a sporadically occurring condition, a serious effort to determine etiology may require both reactive and prospective approaches. Collaboration with public health, health professionals, law enforcement and persons who use illegal drugs, would facilitate the early identification of cases to enable timely and complete follow-up including obtaining heroin samples. A pre-arranged process for transporting and testing implicated heroin samples may allow identification of the contaminant and therefore prevent further cases. Purity and contaminant sampling programs for street drugs could also be considered. It is therefore important to ensure key stakeholders are aware of these findings and the association of leukoencephalopathy and heroin smoking.

## Competing interests

The authors declare that they have no competing interests.

## Authors' contributions

JAB oversaw and gave input at all levels of the investigations. ML and LC followed up the cases in Vancouver. RS and LS followed up the cases in Victoria. LC wrote a report on Vancouver cases. RS wrote the first draft of the paper. NA performed an updated literature review and edited the paper. SDS provided clinical expertise. All authors have read, given input and approved the final manuscript.
